# Comprehensive Treatment of Hematological Patients with SARS-CoV-2 Infection Including Anti-SARS-CoV-2 Monoclonal Antibodies: A Single-Center Experience Case Series

**DOI:** 10.3390/curroncol29040188

**Published:** 2022-03-26

**Authors:** Göran Ramin Boeckel, Silke Dorothea Hölscher, Christin Bürger, Torid Jacob, Carolin Krekeler, Evgenii Shumilov, Christian Reicherts, Annalen Bleckmann, Georg Lenz, Richard Vollenberg, Phil-Robin Tepasse

**Affiliations:** 1 Department of Medicine B for Gastroenterology, Hepatology, Endocrinology and Clinical Infectiology, University Hospital Muenster, 48149 Muenster, Germany; silkedorothea.hoelscher@ukmuenster.de (S.D.H.); christin.buerger@ukmuenster.de (C.B.); torid.jacob@ukmuenster.de (T.J.); richard.vollenberg@ukmuenster.de (R.V.); 2 Department of Medicine D for Nephrology and Rheumatology, University Hospital Muenster, 48149 Muenster, Germany; 3 Department of Medicine A (Hematology, Oncology, Hemostaseology and Pulmonology), University Hospital Muenster, 48149 Muenster, Germany; carolin.krekeler@ukmuenster.de (C.K.); evgenii.shumilov@ukmuenster.de (E.S.); christian.reicherts@ukmuenster.de (C.R.); annalen.bleckmann@ukmuenster.de (A.B.); georg.lenz@ukmuenster.de (G.L.)

**Keywords:** SARS-CoV-2, COVID-19, hematologic malignancies, cancer, antibody therapy, casirivimab, imdevimab, bamlanivimab

## Abstract

**Simple Summary:**

SARS-CoV-2 causes coronavirus disease (COVID-19) and poses a global health burden. Treatment strategies remain limited and mainly consist of dexamethasone; antiviral agents, such as remdesivir and molnupiravir; and SARS-CoV-2-specific monoclonal antibodies (mABs), including bamlanivimab, casirivimab/imdevimab combination, and, most recently, sotrovimab. SARS-CoV-2 mABs target specific proteins on the viral surface. Patients with hematologic malignancies represent an especially vulnerable cohort. Evidence for the use of SARS-CoV-2 mABs in these patients is scarce, creating an imbalance between urgently needed therapeutic strategies and a lack of randomized controlled trials. In this study, we summarize the characteristics of tolerability and clinical benefits of SARS-CoV-2 mAB application in patients with COVID-19 and hematologic malignancies admitted to our university hospital in Germany. We hope to provide some evidence that may contribute to the management of patients with hematologic malignancies in a world where COVID-19 remains a risk.

**Abstract:**

Patients with hematologic malignancies are at high risk of exacerbated condition and higher mortality from coronavirus disease 2019 (COVID-19). Bamlanivimab, casirivimab/imdevimab combination, and sotrovimab are monoclonal antibodies (mABs) that can reduce the risk of COVID-19-related hospitalization. Clinical effectiveness of bamlanivimab and casirivimab/imdevimab combination has been shown for the Delta variant (B.1.617.2), but the effectiveness of the latter treatment against the Omicron variant (B.1.1.529) has been suggested to be reduced. However, the tolerability and clinical usage of severe acute respiratory syndrome coronavirus 2 (SARS-CoV-2)-specific mABs in patients with hematologic malignancies are less specified. We present a retrospective case series analysis of all SARS-CoV-2-infected patients with hematologic malignancies who received SARS-CoV-2-specific mABs at our facility between February and mid-December 2021. A total of 13 COVID-19 patients (pts) with at least one malignant hematologic diagnosis received SARS-CoV-2-specific mABs at our facility, with 3 pts receiving bamlanivimab and 10 pts receiving casirivimab/imdevimab combination. We observed SARS-CoV-2 clearance in five cases. Furthermore, we observed a reduction in the necessity for oxygen supplementation in five cases where the application was administered off-label. To the best of our knowledge, we present the largest collection of anecdotal cases of SARS-CoV-2-specific monoclonal antibody use in patients with hematological malignancies. Potential benefit of mABs may be reduced duration and/or clearance of persistent SARS-CoV-2 infection.

## 1. Introduction

Severe acute respiratory syndrome coronavirus 2 (SARS-CoV-2) causes coronavirus disease 2019 (COVID-19), posing tremendous challenges to patient care. Patients with hematologic malignancies represent an especially vulnerable cohort within the ongoing pandemic. Since the COVID-19 emergence, steady progress has been achieved in treatment strategies aimed at preventing or mitigating severe and critical COVID-19.

Current treatment approaches aim to reduce overactivated host immune response to SARS-CoV-2 itself as well as invasion of the latter in the host. Dexamethasone became a part of evidence-based treatment at the beginning of the pandemic in severe and critical COVID-19 [1]. While the doses are being discussed [2,3], an evidence-based statement concerning patients with hematologic diseases is lacking. Remdesivir, an inhibitor of the viral RNA-dependent RNA polymerase, was found to be the first efficacious antiviral agent against SARS-CoV-2, shortening the time to recovery in adults (10 days compared to 15 days for placebo) [4]. Since February 2021, the administration of monoclonal antibodies (mABs), namely bamlanivimab (LY-CoV555) [5] and casirivimab/imdevimab combination (REGN-CoV-2) [6], have been part of the treatment options against COVID-19 in Germany. In this scenario, the application of mABs in patients with hematologic and oncologic malignancies is of special interest given they are prone to exacerbated condition and thus higher mortality rate from COVID-19 [7,8].

Initially, mABs were available for hospitalized patients only within a national emergency program. Bamlanivimab, the first anti-SARS-CoV-2 mAB, targets the surface spike glycoprotein of SARS-CoV-2. It was derived from a patient who had recovered from COVID-19. Its use was authorized for mild to moderate COVID-19, and the treatment has been shown to reduce the risk of hospitalization or emergency department visits (1.6% vs. 6.3% for placebo). However, the authorization was later revoked for single use [5,9]. Its effectiveness in reducing COVID-19-related hospitalizations, especially for older (>65) and obese (BMI > 35 kg/m^2^) patients, has been demonstrated [10]. In contrast to bamlanivimab, casirivimab/imdevimab combination treatment involve recombinant human mABs. They are directed against distinct, nonoverlapping epitopes on the spike protein, and their combined use has been found to reduce the risk of any-cause death and viral load more rapidly than placebo [6]. The distinct epitope targeting feature is of special importance as it reduces the risk of occurrence of escape variants of the virus. The treatment was initially authorized for mild to moderate COVID-19 but was later extended as postexposure prophylaxis and then (in November 2021 when the predominant variant was Delta B.1.617.2) as preexposure prophylaxis for patients who are not expected to achieve an adequate immune response to complete vaccination. However, the casirivimab/imdevimab treatment has been shown to have insufficient effectiveness against the Omicron variant (B.1.1.529). Concerning the latter variant, considerable humoral immune evasion was shown for both bamlanivimab and casirivimab/imdevimab despite distinct epitope targeting [11]. Within the range of the national prescribing instructions of the German Federal Ministry of Health and Internal Hospital regulations and in accordance with the hospital’s COVID-19 Expert Committee guidance, we started administering mABs in selected cases. The experience with anti-SARS-CoV-2 mAB application in patients with hematologic malignancies is scarce. Here, we demonstrate the characteristics of tolerability and clinical usage of anti-SARS-CoV-2 mAB application in patients with COVID-19 and hematologic malignancies admitted to our university hospital in Germany.

## 2. Materials and Methods

### 2.1. Design

This retrospective single-center case series enrolled all patients with SARS-CoV-2 infection and hematological malignancy admitted to our center, with the patients receiving either bamlanivimab or casirivimab/imdevimab combination treatments. All patients with hematologic malignancy were identified by confirmed diagnosis in the medical records provided either by the patients upon admission or from the electronic database (if previous visits had occurred). SARS-CoV-2 positivity was assessed by nasopharyngeal and oropharyngeal swabs, which were tested for 2019-nCoV nucleic acid by reverse transcription polymerase chain reaction (RT-PCR). A diagnosis of COVID-19 was made following Robert Koch Institute guidelines if clinical indicators (acute respiratory symptoms of any severity, loss of sense of smell, olfactory sense, or disease-related death) and positive PCR test were found [12]. mABs were administered after intensive interdisciplinary decision-making and, in some cases, as part of an individual healing attempt or off-label administration. Decisions were made on a case-by-case basis with therapeutic and preemptive intent.

### 2.2. Data Collection

Laboratory and radiological information were available in the electronic records. We used a standardized electronic data collection sheet to find relevant information for each patient. Due to the retrospective nature of our case series, only available data in medical records were used, which, by the nature of collection, were inhomogeneous. GraphPad Prism 9 software was used to depict data points.

## 3. Results/Presentation of Cases

Between February 2021 and mid-December 2021, a total of 13 patients with hematologic malignancies and SARS-CoV-2 infection received anti-SARS-CoV-2 mAB treatment at our facility. All patients were either symptomatic for COVID-19 or amidst therapies of the individual hematologic malignancy with presumed prolonged viral clearance time and a higher likelihood of severe COVID-19. Thus, the cohort represented real-world cases, including hospitalization with and because of COVID-19. The median age was 57 (range: 21–77). The median hospitalization time was nine days (range 3–45). The most relevant patient characteristics and data regarding treatment are summarized in Table 1.

If possible, follow-up data was acquired from the internal hospital records. To our knowledge, no patient has died, although one was ultimately switched to best supportive care (BSC) due to progression of the underlying hematologic malignancy and discharged home with palliative intent (case #9). The median follow-up from admission to our facility was 93 days (range: 4–385). Four patients were lost to follow-up/had no further electronically documented visits at our facility. For six patients, SARS-CoV 2 PCR swabs from consecutive visits to our hospital were found in the electronic records (documented follow-up median was 100 days (range 23–382)). As admission policy of our hospital requires patients to present a negative PCR upon each visit, negative PCRs for all patients who returned to our hospital could be assumed.

Below, we present a more detailed description of our experience with anti-SARS-CoV-2 mAB treatment summarized by underlying hematologic disease. We selected the four most insightful and interesting cases (#7,9,10, and 13) for the main part of this manuscript. A more detailed description of the remaining cases is presented in the Supplementary Materials File S1.

### 3.1. Lymphoma

#### 3.1.1. Case 7: 53 y/o, Female, B-Cell Chronic Lymphocytic Leukemia (B-CLL)

A 53-year-old patient with a 10-year history of B-CLL was admitted to our SARS-CoV-2 unit as a transfer from another hospital due to severe COVID-19 pneumonia. At presentation, the patient had been in complete remission without CLL treatment for 10 years (Table 1).

**Figure 1:**
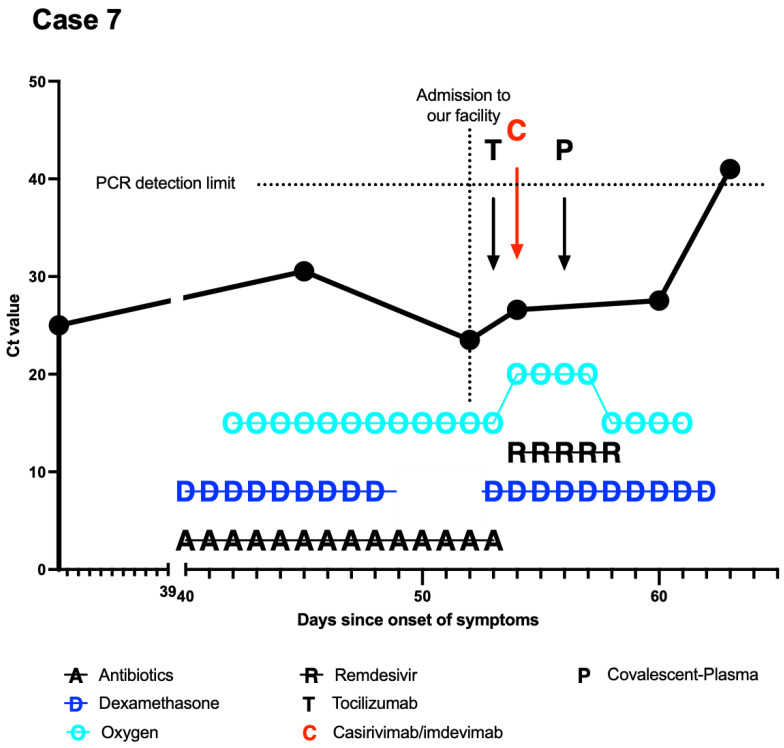
Course of treatment of case 7. *X*-axis was modified to emphasize the period at our facility. Day 0 (*Y*-axis) signifies the onset of symptoms. PCRs with nonmeasurable results were set at 41 (above detection limit). Continuous/repeated applications are connected by a line. The patient was transferred to our facility with an increasing need for oxygen supplementation. Roughly a week after the described multiple interventions, the effects were seen with increasing Ct values.

At the time of transfer, the patient had already been hospitalized for two weeks preceding double vaccination (Table 1) and needed oxygen supplementation. In sum, the initial SARS-CoV-2 diagnosis had been made more than seven weeks before transfer to our hospital. At the transferring hospital, she had already received dexamethasone (6 mg for 10 days) as well as an anti-infective therapy with piperacillin/tazobactam, meropenem, and caspofungin. Bronchoalveolar lavage did not point to superinfection. Chest CT scan showed typical COVID-19 infiltrations. Upon admission to our clinic, PCR showed a Ct value of 23 (Figure 1), and the patient received a course of high-dose dexamethasone (20 mg for five days, followed by 10 mg for five days) as well a single dose of tocilizumab (600 mg) because of suspected hyperinflammation after persistently elevated inflammatory markers, even after completion of low-dose dexamethasone. One day after admission (day 53 since the onset of symptoms), casirivimab/imdevimab was administered, followed by remdesivir. The decision for this off-label application so late in the infection was made after interdisciplinary consultation. The therapy was well tolerated.

Due to persisting high-flow oxygen therapy, the patient additionally received SARS-CoV-2 convalescence plasma 2 days post mAB application within a national trial. Subsequently, oxygen therapy was steadily reduced to nasal cannula and finally discontinued 7 days after application of anti-SARS-CoV-2 mABs. Both the symptoms and the blood work improved, and the patient was discharged 25 days after admission (63 days after onset of symptoms).

#### 3.1.2. Case 9: 54 y/o, Male, Diffuse Large B-Cell Lymphoma (DLBCL) following Multiple Prior Treatments

A 54-year-old unvaccinated man with DLBCL was transferred from a peripheral hospital because of newly diagnosed meningeosis lymphomatosa and persistent SARS-CoV-2 infection.

DLBCL had been present in the left kidney, gastric area, and frontal mediastinum. The patient had undergone chemotherapy (four cycles of R-CHOP, followed by four cycles of CHOEP-14; Table 1) two weeks before testing positive for SARS-CoV-2 and had mild COVID-19. After the quarantine had been lifted because of high Ct value in PCR swabs, he presented to the initial clinic with peripheral facial nerve paralysis on the right. After ruling out stroke, lumbar puncture revealed pleocytosis (4253/3 cells (<4)), consistent with meningeosis lymphomatosa (FACS). Cerebral and spinal MRI confirmed the affection of several cranial and spinal nerves.

**Figure 2:**
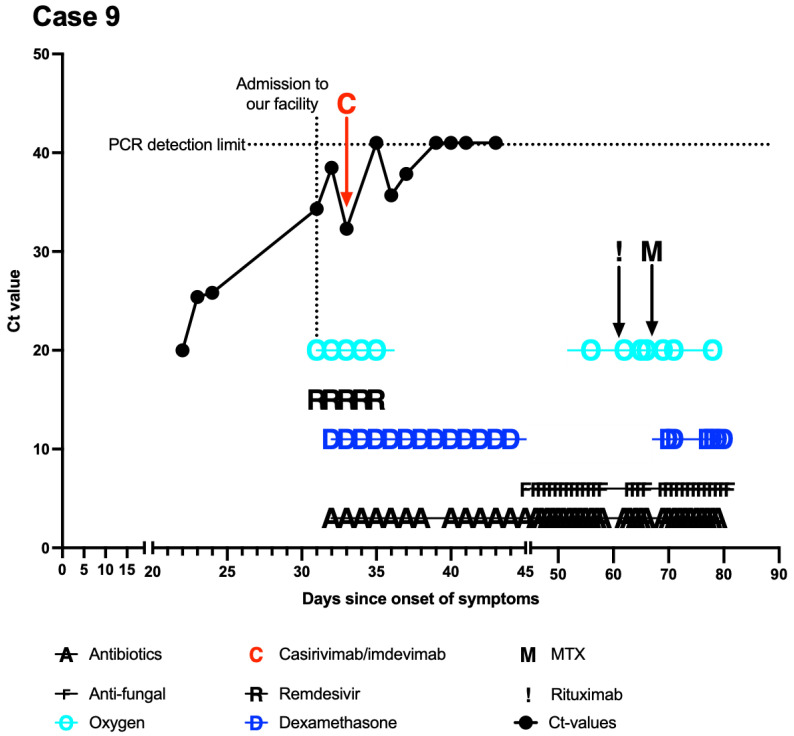
Course of treatment of case 9. *X*-axis was modified to emphasize the period at our facility. Day 0 (*Y*-axis) signifies the onset of symptoms. PCRs with nonmeasurable results were set at 41 (above detection limit).

Further diagnoses included diabetes mellitus type 2, arterial hypertension, and coronary vessel disease with myocardial infarction four years before presentation. Subsequent SARS-CoV-2 PCR swabs revealed undulating low Ct values (Figure 2). The patient was again quarantined and transferred to our SARS-CoV-2 unit.

Upon presentation at our clinic, the patient presented no COVID-19 symptoms. However, in addition to peripheral facial nerve paralysis, a diagnosis of bilateral hypoglossal nerve paralysis was made. Because of markedly elevated inflammatory markers and progressive need for oxygen supplementation (up to 8 L via nasal cannula), superinfection was suspected. A chest CT scan revealed findings consistent with aspiration pneumonia and showed mild left pleural effusion. Calculated antibiotic treatment with piperacillin/tazobactam was initiated. Due to persistently low Ct values and progression of DLBCL, a therapy with dexamethasone and remdesivir was started. Subsequently, casirivimab/imdevimab was started (roughly 30 days after initial onset of symptoms). The treatment was well tolerated, and Ct values began to increase. At the last follow-up, roughly 45 days after the initial onset of symptoms (18 days after casirivimab/imdevimab), the patient was de-isolated after four consecutive negative PCR swabs. Following further rapid progress of DLBCL, the therapeutic concept was changed to BSC. The patient was discharged home with mobile nursing service and a palliative care team.

#### 3.1.3. Case 10: 59 y/o, Male, DLBCL, Multiple Prior Treatments

A 59-year-old man with a 13-year history of nasopharyngeal and oropharyngeal DLBCL and multiple prior therapies was transferred from a peripheral hospital with severe COVID-19-pneumonia requiring oxygen therapy.

The patient had been vaccinated twice (BioNTech/Pfizer), with the latest dose 24 days before the onset of symptoms (loss of appetite, weight loss, and deterioration of general state). Prior oncological treatments included four therapy lines, including HDCT/ASCT, and most recently, radiotherapy of the tonsil three months before SARS-CoV-2 infection. According to the most recent CT, the patient was in CR at the time point of COVID-19 infection. Due to moderately increased CRP (27.7 mg/L (<5)) and pancytopenia (leucocytes 0.9/nL, hemoglobin 9.6 g/dL, and thrombocytes 49/nL), an antibiotic treatment alongside stimulation with G-CSF had been initiated in the transferring hospital. Despite the abovementioned means, no improvement was documented.

**Figure 3:**
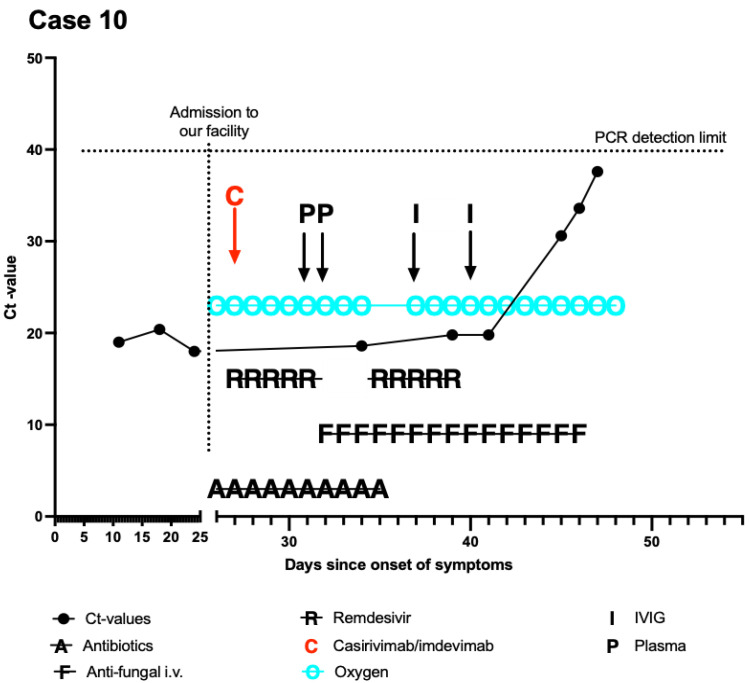
Course of the disease of case 10. *X*-axis was modified to emphasize the period at our facility. Day 0 (*Y*-axis) signifies the onset of symptoms. PCRs with nonmeasurable results were set at 41 (above detection limit).

Upon transfer to our SARS-CoV-2 unit, the Ct value had remained low (19) (Figure 3), while inflammatory markers were markedly increased (CRP 10 mg/dL (<0.5), ferritin 7872 µg/L (<400), and interleukin-6 61 pg/mL (<7)). Furthermore, a severe secondary immunoglobulin deficiency was detected (IgG 248 mg/dL (700–1600), and IgA 16 mg/dL (70–400), IgM 6 mg/dL (40–230)). Blood tests for EBV and CMV (PCR) remained negative. Cellular immune status revealed severe lymphopenia (90/µL (1200–3000)) with decreased B- and T-lymphocytes. Consequently, titers against SARS-CoV-2 were negative despite vaccination. Because of persisting anemia and thrombocytopenia, the recommendation for bone marrow biopsy after full clearance was made. After ruling out vitamin deficiency and due to multiple chemotherapies, a differential diagnosis of therapy-induced myelodysplastic syndrome (tMDS) was considered.

On day 26 after the onset of symptoms, the patient received casirivimab/imdevimab alongside remdesivir. The treatment was well tolerated. Additionally, the patient received SARS-CoV-2 convalescence plasma 4 days post mAB application within a national trial. Simultaneously, stimulation with G-CSF was continued, and immunoglobulins were substituted. Because of the ongoing need for oxygen supplementation with severe tachypnea and exertional dyspnea, chest CT scan was repeated. Fungal pneumonia could not be ruled out, and the patient was started on voriconazole. In further CT scans, the presumed fungal lesions were regressive, suggesting fungal pneumonia as one important differential diagnosis for the prolonged recovery of this patient. In parallel, inflammatory markers declined, and oxygen supplementation was reduced. The patient showed slow but steady improvement. The patient was discharged virus-free after a total of 45 hospitalization days.

### 3.2. Acute Leukemia

Case 13, AML (Acute Myeloid Leukemia), Newly Diagnosed

**Figure 4:**
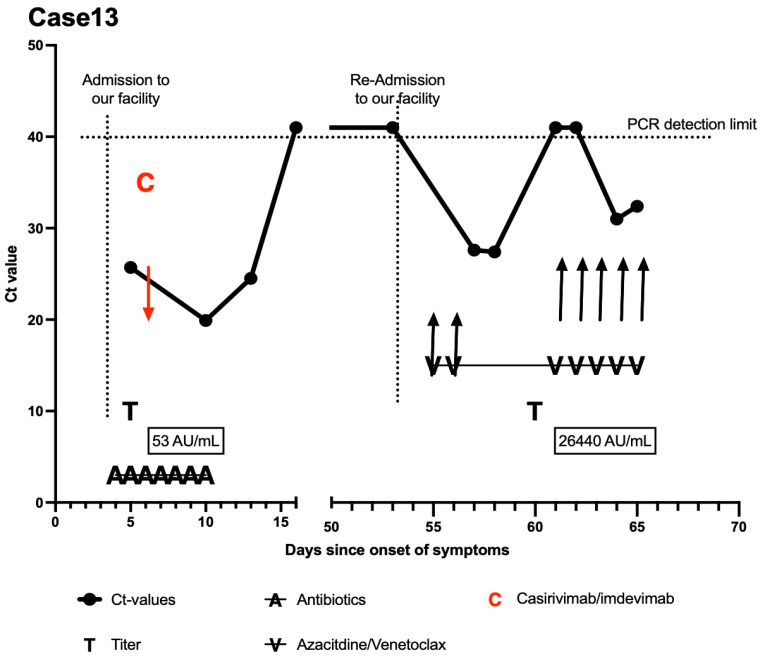
Course of the disease of case 13. *X*-axis was modified to emphasize the period at our facility. Day 0 (*Y*-axis) signifies the onset of symptoms. PCRs with nonmeasurable results were set at 41 (above detection limit). The patient received casirivimab/imdevimab and was discharged to home quarantine. He presented several negative PCR swabs before readmission and started systemic therapy with azacitidine and venetoclax. Upon routine testing, low Ct values consistent with reactivation (a reinfection was ruled out by sequencing) were observed, quickly resolving after pausing azacitidine and venetoclax. After restart of therapy, the Ct values again decreased. Note: more than 50 days after casirivimab/imdevimab infusion (anti-SARS-CoV-2 spike protein IgG > 40,000 AU/mL at our facility), the patient had remaining anti-SARS-CoV-2 spike protein IgG titer of 26,440 AU/mL.

A 55-year-old man who had recently (3 weeks prior) been diagnosed with de novo AML presented with fever, dyspnea, and cough. The patient claimed to have had a SARS-CoV-2 infection several months ago (anti-SARS-CoV-2 spike protein IgG antibody titer: 53.4 AU/mL (Figure 4)) and was therefore unvaccinated. As an additional risk factor of severe SARS-CoV-2 infection, he had a history of chronic kidney disease (CKD G4 A3), arterial hypertension, and several myocardial infarctions (the most recent being 2 months prior to presentation) and was thus receiving dual antiplatelet therapy. One week before symptom onset, he was scheduled to receive azacitidine and venetoclax therapy, which was not begun following SARS-CoV-2 infection. His laboratory showed pancytopenia and CRP elevation (20 mg/dL (<0.5)). Chest CT scan showed bipulmonary, multifocal ground-glass opacities compatible with both SARS-CoV-2 pneumonia and local AML manifestations. A calculated antibiotic treatment with piperacillin/tazobactam was started (DD: superinfection). Acute chronic kidney injury was attributed to the rising levels of uric acid (10.8 mg/dL (3.6–6.5)). To prevent a severe course of COVID-19, an interdisciplinary decision was made to administer casirivimab/imdevimab (Figure 4). The treatment was applied 5 days after the onset of symptoms and was well tolerated. The patient required no more oxygen therapy. He was released to home quarantine and closely monitored via laboratory controls.

After the clearance of the virus (two negative nasopharyngeal swabs), the patient was readmitted for the start of azacitidine and venetoclax. In routine testing 2 days after initiation of the therapy, a Ct value of 27.6 was found, requiring the patient to be readmitted to our SARS-CoV-2 surveillance unit. Azacitidine and venetoclax were paused. The patient was entirely asymptomatic; however, due to the emerging Omicron variant (B.1.1.529), both reinfection with Omicron and a reactivation of the previous viral variant (Delta variant B.1.617.2) were considered. Sequencing showed a persistent Delta infection. Roughly one month after initial treatment with casirivimab/imdevimab, antibody titers remained high at 26,440 AU/mL. Further swabs confirmed falling CT values. The patient was closely observed for the occurrence of symptoms and was started on a daily testing routine. After testing negative for SARS-CoV-2, azacitidine and venetoclax were continued. Once again, 3 days after the restart of therapy, PCR results turned out to be positive. The patient ultimately discharged himself from the hospital against medical advice.

The remaining nine patients received SARS-CoV-2 mABs due to symptomatic SARS-CoV-2 infection (eight out of nine pts) or preemptively due to anticipated severe course and/or urgent need for rapid viral clearance (case #3). After the application of SARS-CoV-2 mABs, oxygen supplementation could be discontinued in both cases who had intermittently required it earlier (cases #1 and 5). Symptom alleviation was achieved in all eight symptomatic pts. Furthermore, viral clearance was observed in case #11. However, the majority of these cases presented with mild symptoms, and viral follow-up was not observed/measured due to early release to home quarantine.

## 4. Discussion

We present 13 cases of SARS-CoV-2-infected patients with hematologic malignancies that received SARS-CoV-2-specific mAB treatment at our facility. To our knowledge, this is the largest case series summarizing the baseline characteristics, clinical data, clinical presentation, and, where possible, outcome of these special patients.

A search of the NCBI library on 15 January 2022 with a filter for “case reports” and search terms “COVID-19”, “cancer”, “monoclonal antibody therapy” “bamlanivimab”, and “casirivimab” retrieved 58 results, the majority of which described the use of immune checkpoint inhibitors for COVID-19 or the use of tocilizumab or convalescence plasma. Three case reports were found (Table 2) describing the use of SARS-CoV-2-specific mABs in hematologic malignancies [13].

The first successful clearance after SARS-CoV-2-specific mAB treatment was described in a follicular lymphoma (FL) patient who had undergone treatment with obinutuzumab (anti-CD20 mAB) and bendamustine (the latter leading to delayed T-lymphocyte reconstitution).

The authors described persistent virus detection (300 days) despite several therapies, including remdesivir, convalescent plasma, and IVIGs [13]. Interestingly, initial treatment with casirivimab/imdevimab merely resulted in an incomplete response, and a second application 6 weeks later finally led to full clearance. Of note, the concentrations used were much higher (4 g each antibody) than in our cohort. Similar to our observation, the authors argued strongly in favor of establishing specific guidelines for the treatment of this distinct population discussed here, i.e., patients with hematologic malignancies.

Taha et al. described the clinical courses of two lymphoma patients: a patient with FL treated with multiple therapies, including CD20/anti-CD3 bispecific antibody, and a patient with anti-PD-L1 and CLL under ibrutinib treatment [14]. After a long period without viral clearance (>200 days), casirivimab/imdevimab treatment in combination with remdesivir led to viral clearance after six days (and in the case of the CLL patient, 3 days).

Similarly, three of our cases (#7,9, and 10) presented with prolonged viral detection (range: 26–53 days) before mAB application and were likewise suffering from underlying lymphoid malignancies. A definitive SARS-CoV-2 clearance was observed within 10 days post mAB application.

Concerning the combination of bamlanivimab and etesevimab, Saultier et al. described successful application in a patient with newly diagnosed leukemia [15]. Viral clearance was achieved 10 days after treatment, and consecutive tests of mAB titers showed decreased but still detectable titer even after two months. While their patient did not present respiratory symptoms, our case (#13) was initially symptomatic and presented with more severe comorbidities. Analogously, persisting titers of SARS-CoV-2 mAB were detected more than 55 days after administration (Figure 3). Thus, mABs seem to persist longer in distinct cases.

Until mid-2021, there had been mainly six randomized controlled trials (RCTs) providing results from more than 17,000 participants considering the effectiveness and safety of SARS-CoV-2-neutralising mABs for treating patients with COVID-19 [16]. While four studies mostly focused on nonhospitalized patients, Kreuzberger et al. [16] identified and assessed two studies evaluating the use of mABs in hospitalized patients, namely bamlanivimab [17] and casirivimab/imdevimab combination [18]. The authors concluded the evidence to be insufficient to draw meaningful conclusions concerning the value of anti-SARS-CoV-2 mABs. The main criticism concerned the lack of overall evidence, but the evidence provided also did not show significant differences in the main categories of survival, symptom alleviation, hospitalization time, or side effects.

More than 30 trials are currently ongoing. Just recently, O’Brien et al. outlined that the use of subcutaneous casirivimab and imdevimab in asymptomatic SARS-CoV-2 PCR-positive people living with an infected household contact reduced the incidence of symptomatic COVID-19 over 28 days in a randomized trial with 314 participants [19]. These are encouraging results, although there is a potential shortcoming of bias as the cohort was not particularly vulnerable per se and no Omicron cases were included (due to prior enrollment).

In general (for all patients), German general practice guidelines recommend the use of SARS-CoV-2-specific mABs in patients with IgG seronegativity and, at most, low-flow oxygen [20]. It is worth noting that EMA authorized the use of casirivimab/imdevimab (discrepantly) for patients who do not require supplemental oxygen [21]. The general practice guidelines do not make specific claims about cancer patients.

There are several caveats to our study. Firstly, by the nature of a case series, no statistics beyond mere numerical description can be applied, thus limiting the generalizability. Secondly, despite offering a wide variety of several cases, the group/risk factor “hematologic neoplasia” is much too vast and inhomogeneous to be even covered by the greatest RCT. Thirdly, during the pandemic, clinical decisions have been deeply affected by political decisions and economical availability. Thus, both the patient group, i.e., according to underlying disease, and the applied antibody treatment is heterogenous, further impeding comparability. While vaccination had been a matter of availability at the beginning of the observed period, it has become a matter of personal choice at present (and, soon, maybe governmental mandated (in Germany)). With two antibodies used in our study and a third (sotrovimab) on the brink of general availability (in Germany), not only the virus variants but also the treatment options make longitudinal comparisons difficult. Case series have been used in other SARS-CoV-2 subcohort investigations to provide more insights beyond RCTs, such as myocarditis [22] and diabetes [23].

### Perspective

While this manuscript was being drafted, a new mutated SARS-CoV-2 virus variant, named Omicron, has emerged and been characterized as variant of interest (VOI) and variant of concern (VOC). Major knowledge gaps regarding VOC Omicron urgently need to be filled [24]. There are currently (while in preprint, December 2021) concerning in vitro data demonstrating resistance to casirivimab and imdevimab. This shows that virus genotyping may be necessary before antibody administration and also underlines the need for variant-specific mAB agents for the Omicron and other emerging variants of concern [25].

## 5. Conclusions

Our study presents real-world experience on the application of SARS-CoV-2 mAB treatment in patients with hematologic malignancies. To the best of our knowledge, this is currently the largest collection of anecdotal cases of SARS-CoV-2-specific monoclonal antibody use in patients with hematologic malignancies. Case-based retrospective approaches can be useful to generate hypotheses. Several open research questions are unanswered and/or in the process of answering. As we learn more about the intricacies of prevention, risk stratification, and treatments, reflection on currently treated patients in need of direct intervention should be made critically with the careful and observing eyes of clinicians and scientists alike.

The main learning points we can summarize are as follows:

(1) We observed clinical courses suggesting that SARS-CoV-2-specific monoclonal antibodies (bamlanivimab, casirivimab, and imdevimab) are well tolerated for the treatment of SARS-CoV-2 infection in patients with hematologic malignancies.
(2) The overall evidence for use of SARS-CoV-2-specific mABs is scarce. Studies are urgently needed to assess the clinical effectiveness of such therapy in patients with hematologic malignancies (especially if no/minor vaccination response is observed).
(3) Potential benefit of application may be reduced duration of disease and/or clearance of persistent SARS-CoV-2 infection.
(4) Close attention should be paid to the baseline characteristics (variant, vaccination status, time courses, and prior and additional treatments) to generate further hypotheses in an otherwise bleak landscape of clinical evidence.
(5) Superinfections and other respiratory viruses should not be forgotten (case #8).

## Figures and Tables

**Table 1:** Characteristics of SARS-CoV-2-infected patients with hematologic malignancies receiving mAB therapy.

Disease, Initial Diagnosis, Mo/YY (Age at Infusion Time, Sex) Stage/Point in Therapy	Variant Immunization Antibody Titer *	Antibody (mg) Day since Symptom Onset	Hospitalization Oxygen Requirement Outcome
Multiple Myeloma			
Case 1: MM, IgG lambda,11/08 (59 y/o, male) Maintenance therapy lasted 6 d prior to symptom onset	N/d Unknown Not determined	Bamlanivimab 700 Day 4 (off-label)	9 days Nasal canula 1–3 L Released virus-free
Case 2: MM, IgG lambda, 08/14 (53 y/o, male) Maintenance therapy: daratumumab	N/d BioNTech 2× (>6 months before onset) Anti-SARS-CoV-2 spike protein IgG: 177.1 AU/mL	Casirivimab 1200/imdevimab 1200 Day 7 (off-label)	7 days No oxygen required Home quarantine
Case 3: MM Type, IgG lambda, 06/21 (63 y/o, male) Stem cell apheresis stimulation on day 1 before symptom onset; four induction cycles completed (Dara-VTD)	B.1.617.2 (Delta) AstraZ. 2× (4–5 months before onset) negative	Casirivimab 1200/imdevimab 1200 Day 1 (off-label)	3 days No oxygen required Home quarantine
Case 4: MM Kappa, 05/21 (61 y/o, male) Stem cell apheresis with cyclophosphamide induction (day 18 before symptom onset); four induction cycles completed	B.1.617.2 (Delta) BioNTech 2× (6 mo. before onset) Anti-SARS-CoV-2 spike protein IgG: 78.7 AU/mL	Casirivimab 1200/imdevimab 1200 Day 2 (off-label)	3 days No oxygen required Home quarantine
Lymphoma other than MM			
Case 5: B-CLL, 11/03 (63 y/o, male) Asymptomatic, no therapy	B.1.617.2 (Delta) BioNTech 3× (24 days before onset) Negative	Casirivimab 600/imdevimab 600 Day 14 (off-label)	5 days Nasal canula 2 L Home quarantine
Case 6: B-CLL, 09/97 (77 y/o, male) Several prior therapies; venetoclax since 2 years prior to symptom onset	B.1.617.2 (Delta) 3× no more information available Negative	Casirivimab 1200/imdevimab 1200 Day 5 (off-label)	10 days No oxygen required Home quarantine
Case 7: B-CLL, 04/11 (53 y/o, female) No therapy, CR, FCR 07-11/11	N/d most likely B.1.617.2 (Delta) 2× no more information available Negative	Casirivimab 1200/imdevimab 1200 Day 53 (off-label)	25 days High-flow oxygen therapy Home quarantine
Case 8: FL, 09/17; DLBCL, 10/14 (57 y/o, female) HD-BEAM >1 year prior to symptom onset; Maintenance therapy of rituximab until 8 months before symptom onset	N/d most likely B.1.617.2 (Delta) BioNTech 2× (1 day before onset) Negative	Casirivimab 1200/imdevimab 1200 Day 3 (off-label)	5 days No oxygen required Home quarantine
Case 9: DLBCL, 09/21, cerebral manifestation (54 y/o, male) CHOEP 14 fourth cycle ≈ day 15 before symptom onset	B.1.1.7 (Alpha) Not vaccinated Negative	Casirivimab 1200/imdevimab 1200 ≈Day 30 (off-label)	31 days Nasal canula 8 L BSC, released virus-free
Case 10: DLBCL, 06/08 (59 y/o, male) Multiple (R-CHOP, R-DHAP, 2× HD-BEAM) most recent therapy: irradiation of tonsil 3 months prior to symptom onset	AY.9.2 (Delta) BioNTech 2× (24 days before onset) Negative	Casirivimab 1200/imdevimab 1200 Day 26 (off-label)	45 days Nasal canula 8 L Released virus-free
Acute Leukemia			
Case 11: AML, NPM-1 mut, 12/20 (21 y/o, male) Daunorubicin/cytarabine/midostaurin 1 months before symptom onset	N/d, most likely B.1.1.7 (Alpha) Unknown Not determined	Bamlanivimab 700 Day 4 (off-label)	14 days No oxygen required Released virus-free; AML relapse 10 months later
Case 12: AML MDS, 03/19 (52 y/o, female) Second allogeneic SCT 7 months before symptom onset; maintenance with sorafenib	B.1.1.7 (Alpha) Unknown Negative	Bamlanivimab 700 Day 7 (off-label)	7 days No oxygen required Home quarantine; AML relapse 2 months later
Case 13: AML with maturation, 09/21 (55 y/o, male) No therapy at present; earlier on azacitidine/venetoclax	B.1.617.2 (Delta) Not vaccinated Anti-SARS-CoV-2 spike protein IgG: 53.4 AU/mL (prior infection several months ago)	Casirivimab 1200/imdevimab 1200 Day 5 (off-label)	11 days No oxygen required Left hospital against medical advice; undulating Ct values

ID = initial diagnosis; N/d = not determined; Mo = month; YY = year; CLL = chronic lymphocytic leukemia; FL = follicular lymphoma; MM = multiple myeloma; DLBCL = diffuse large B-cell lymphoma; CR = complete remission; Dara-VTD = daratumumab, Velcade (bortezomib), thalidomide, dexamethasone; FCR = fludarabine, cyclophosphamide, rituximab; HD-BEAM = high-dose BCNU, etoposide, Ara-C (cytarabine), melphalan; CHOEP = cyclophosphamide, doxorubicin (hydroxydaunorubicin), vincristine (Oncovin^®^), etoposide, prednisolone; R-CHOP = rituximab-cyclophosphamide, doxorubicin (hydroxydaunorubicin), vincristine (Oncovin^®^), prednisolone; Ct = threshold cycle; BSC = best supportive care; BioNTech/Pfizer = BNT162b2; Moderna = mRNA-1273 Astra = ChAdOx1-S. * prior to administration.

**Table 2:** Summary of case reports regarding SARS-CoV-2-specific mAB treatment in cancer patients.

Case Report: Hematologic Malignancy	*n* (Patients)	Antibody	Main Point	Ref.
Follicular lymphoma	1	Casirivimab/imdevimab	Persistent SARS-CoV-2 infection (300 days) viral clearance	[13]
Follicular lymphoma Chronic lymphocytic leukemia	2	Casirivimab/imdevimab	Persistent SARS-CoV-2 infection viral clearance	[14]
Ambiguous lineage acute leukemia	1	Bamlanivimab/etesevimab	Co-occurrence of COVID-19 Administration of antileukemic treatment without delay or interruption	[15]

## Data Availability

The data presented in this study are available in this article (and Supplementary Material).

## Supplementary Materials

The following supporting information can be downloaded at: https://www.mdpi.com/article/10.3390/curroncol29040188/s1, File S1: Detailed presentation of cases #1–6, #8, #11, and #12.
