# Supplementary material for: Comprehensive Treatment of Hematological Patients with SARS-CoV-2 Infection Including Anti-SARS-CoV-2 Monoclonal Antibodies: A Single-Center Experience Case Series
Source: Curr Oncol. 2022 Mar 26;29(4):2312–25. doi: 10.3390/curroncol29040188 (PMC9032833; doi:10.3390/curroncol29040188)
Supplement: Supplementary file 1 [file curroncol-29-00188-s001.zip › curroncol-1612199-supplementary.pdf]

Comprehensive treatment of hematological patients with SARS-CoV-2 infection including anti-SARS-CoV-2 monoclonal antibodies: a single center experience case series

Göran Ramin Boeckel, Silke Dorothea Hölscher, Christin Bürger, Torid Jacob, Carolin Krekeler, Evgenii Shumilov, Christian Reicherts, Annalen Bleckmann, Georg Lenz, Richard Vollenberg, Phil-Robin Tepasse

Supplementary Materials:

A) Multiple Myeloma

Maintenance Therapy

Case 1

One of the first patients to receive an antibody treatment at our facility was a 59-year-old man with a history of multiple myeloma (IgG Lambda; initial diagnosis 11/2008) with a diffuse affection of the vertebral column and 70% infiltration of the bone marrow who moreover suffered from heart failure (HFpEF). He received weekly doses of a chemotherapy, which he could not further specify. During a routine swap testing before the chemotherapy application he was tested positive and quarantined. Within 6 days his condition deteriorated and he was admitted to our hospital with fever and dyspnea.

CT-scan revealed typical infiltration as well as a massive pleural effusion. Laboratory results showed hyperferritinemia (2700µg/L [30-400]), CRP-elevation, as well as lymphopenia. Free lambda light chains were massively elevated (1010mg/L [5,7-26,3]).

Oxygen (1-3L nasal cannula) was administered and a course of Remdesivir was started. Pleurocentesis was performed. Four days after onset of symptoms the patient was started on treatment with bamlanivimab within the emergency use program.

The treatment was well tolerated, the oxygen therapy could be discontinued and the patient was released from the hospital 10 days after onset of symptoms with two PCR negative swaps.

Case 2

Several months later, as Germany was in the grip of the delta variant of SARS-CoV-2, a 53-year-old man with symptomatic multiple myeloma (IgG lambda; initial diagnosis 08/2014) was admitted to our facility. Concerning the myeloma, he had received tandem autologous stem cell transplantations (Induction therapy: bortezomib, lenalidomid, dexamethasone, condition therapy: melphalane) and a maintenance therapy with lenalidomid. After disease progression, he was switched to Daratumumab and was currently undergoing monthly maintenance infusions.

He had received two vaccinations six months prior to the infection. The patient had presumably contracted SARS-CoV-2 from a positive co-worker. His chief complaints were fatigue, fever, dry cough and anosmia.

Antibody testing revealed a very low titer 177,1 AU/mL [within our facility titers after application were >40.000AU/mL]. CRP was moderately elevated (3,4mg/dL [<0,5]). Chest x-ray showed findings consistent with inflammatory changes.

Seven days after onset of symptoms the patient started treatment with casirivimab/imdevimab within the German emergency use program, as well as remdesivir.

The treatment was well tolerated, CRP- levels continued to rise (9mg/dL) the day after application, but dropped from then on. The symptoms ceased except for the anosmia and the patient was ultimately release in home quarantine.

Recent diagnosis (Initial oncologic diagnosis during pandemic)

Case 3

A 63-year-old man with multiple osteolytic vertebral fractures, renal impairment and high risk genetics (multiple myeloma IgG lambda; ID 06/2021) was prepared for stem cell aphaeresis after 4 cycles of induction therapy (Daratumumab, Bortezomib, Thalidomide, Dexamethason) at the oncologic ward of our facility. During routine testing he was tested positive for SARS-CoV-2.

Despite two vaccinations (AstraZeneca) negative antibody titers were found. CRP-levels were only mildly elevated. Throughout the patient was asymptomatic, because of the recently applied oncologic therapy and the dire need of accelerated viral clearance to continue his therapy, an interdisciplinary decision was made to offer the patient a treatment with casirivimab/imdevimab. The therapy was well-tolerated and the patient was released into home quarantine the following day.

Case 4

A 61-year-old man with more recently diagnosed multiple myeloma (kappa; initial diagnosis 05/2021) had just completed 4 induction cycles (Daratumumab, Bortezomib, Thalidomide, Dexamethason) and was hospitalized for stem cell apheresis at our facility with cyclophosphamid. He had been released from the hospital shortly before the notice of the above mentioned outbreak in the oncology ward and was traced to be a close contact person of the initially infected patient (case 3). In parallel to PCR testing the patient developed dry cough, fatigue and a fever (38,3°C) and was admitted to our SARS-CoV-2 unit. Laboratory results revealed mildly elevated inflammatory parameters and a very low antibody count was detected (78,7 AU /mL) despite full vaccination (2x BioNTech/Pfizer) 6 months prior to the onset of symptoms. Chest-CT-scans showed no pulmonary abnormalities.

Due to the recent applications of therapy and the presumed weakened immune system the patient was offered an antibody treatment with casirivimab/imdevimab. Shortly after application the patient showed elevated temperatures, however, the symptoms subsided and the temperatures remained within normal range beginning the following day. The patient was released from the hospital into home quarantine.

B) Lymphoma other than multiple myeloma

Case 5 B-CLL, untreated

A 63-year-old man with a 18-year history of previously untreated B-chronic lymphocytic leukemia (B-CLL), as well as secondary antibody deficiency syndrome presented with a two week history of coughing. Since one week, he experienced high fevers (39°C), weight loss (3-4kg in the last 2 weeks) and severe night sweats. He had received three vaccinations with BioNTech/Pfizer (the last 24 days before onset of symptoms).

Chest CT-scan showed bipulmonary, peripheral ground glass opacities, as well as mediastinal lymphadenopathy and splenomegaly. Admission blood work confirmed previously detected leukocytosis (208/nL; predominantly lymphocytosis), as well as anemia (9,8 g/dL) and thrombocytopenia (131/nL). CRP was moderately elevated 3,8 mg/dL [<0,5]. Peripheral oxygen-saturation intermittently dropped below 87%, so 2L of oxygen was supplemented via nasal cannula. Capillary blood gas analysis was stable under oxygen supplementation.

The patient received casirivimab/imdevimab at a dose of 600mg (the official permit for the use had just recently been released and the prior commonly used 1200mg dose packages were split to treat two patients). The night following the application his fever peaked to 40°C.

Due to the secondary antibody deficiency syndrome (IgG 49 mg/dL [700-1600]; IgA 11 mg/dL [70-400]; IgM 6 mg/dL [40-230]) the patient received human immunoglobulins, upon which he presented a temperature of 38,1°C once more and stayed thereafter free of fever. Interestingly, the leukocytosis ameliorated without any signs of tumor lysis syndrome and remained stable around 130/nL.

The patient was discharged in markedly improved general condition into home quarantine. A recommendation for close bloodwork monitoring and previously declined supportive medicine as well as start of B-CLL therapy was expressed.

Case 6 B-CLL, multiple prior treatments, at presentation venetoclax

A three times SARS-CoV-2-vaccinated 77-year-old man with a 24-year history of B-CLL and multiple prior treatments (including Chlorambucil, Fludarabin/Cyclophosphamid, Rituximab-CHOP and most recently Venetoclax [previous 2 years]) presented to the emergency department of our facility with a fever (39°C) and coughing that had begun five days before admission. PCR confirmed SARS-CoV-2 with a Ct-value 17,3. Upon admission his blood work showed pancytopenia (leukocytes 1,6/nL; hemoglobin 9,4g/dL, thrombocytes 23/nL) and significant CRP elevation (9,0mg/dL [<0,5]). ECG showed a first episode of atrial fibrillation, which converted into sinus rhythm after the administration of intravenous Metoprolol.

Venetoclax was paused after consultation with our colleagues from the hematology department and the patient received casirivimab/imdevimab five days after onset of symptoms. The treatment was complemented by remdesivir and ceftazidim. Due to the leukocytopenia the patient received stimulation with G-CSF for six days. Oxygen therapy was not necessary.

His general condition as well as the blood works improved over time and the patient was discharged into home quarantine 10 days after admission with persistently undulating Ct-values.

Case 8 Follicular lymphoma (FL), Diffuse large B-cell lymphoma (DLBCL) multiple prior treatments

A 57-year-old woman with a history of FL and DLBCL presented to our SARS-CoV-2 unit after acquiring a SARS-CoV-2 infection, presumably from her husband.

Hematologically, she had undergone therapy for DLBCL with 8 cycles of Rituximab-CHOEP-14 (cyclophosphamide, doxorubicin, vincristine, etoposide, Dexamethasone) and intrathecal triple therapy in 2015. The diagnosis of follicular lymphoma was made in 2017 and the patient was then treated with Rituximab-DHAP (High dose cytarabin, cisplatinum, dexamethasone). She had received autologous stem cell transplantation (after high-dose BEAM as conditioning regimen) and achieved Rituximab as a maintenance therapy until 8 months before the onset of SARS-CoV-2 symptoms.

Most prominent features of her infection were fever (38,8°C), as well as coughing. She had been immunized twice (BioNTech/Pfizer; the most recent dose had been given a day before onset of symptoms), however, no antibody titers were found. As expected after rituximab-including therapy, the CD19-positive-B-lymphocytes were at 0%. Within the German emergency program, we offered her the application of casirivimab/imdevimab. The patient consented and treatment was initiated on day 3 after onset of symptoms. No adverse reactions were observed. The patient was released 5 days after admission into home quarantine.

Three weeks later the patient was readmitted in reduced general state and with a low fever of 38,1°C. Due to cold like symptoms, productive cough and headache a superinfection or persistent SARS-CoV-2 infection were suspected. Interestingly, she claimed to have been completely free of symptoms in between the two admissions. Ct-value was determined to be 35,9 (no isolation necessary at our facility). In order to rule out differential diagnoses a respiratory virus panel was performed and the patient was tested positive for Parainfluenza 3. The patient was treated symptomatically and could ultimately be released virus- and symptom free from the hospital.

C) Acute Leukemia

Case 11 prior treatments

The first patient at our facility to receive an antibody treatment for COVID-19 was a 21-year-old man with NPM1-positive acute myeloid leukemia (AML).

One month before the initial symptom onset he had finished the second course of an inception chemotherapy with Daunorubicin, Cytarabin and Midostaurin.

An initially asymptomatic SARS-CoV-2 infection was diagnosed in a routine testing upon discharge after surgical treatment of anorectal abscess. He was admitted to our SARS-CoV-2 surveillance ward where he developed mild symptoms. Due to falling Ct-values and after interdisciplinary consultation the patient was offered a treatment with bamlanivimab (within the German emergency use program), followed by Remdesivir. One day after bamlanivimab treatment naso-pharyngeal swap revealed a Ct-value of 21. Starting four days after antibody treatment three consecutive swaps showed no virus. As the parents were also infected and still quarantined, the patient remained in the hospital for a few days.
In the course, the patient underwent consolidation therapy. Due to a relapse (FLT3-ITD; Ratio 0,47) he received gilteritinib and and most recently an allogeneic transplantation.

Case 12 multiple prior treatments

In early march 2021 a 52-year-old woman with a one year history of acute myeloid leukemia with myelodysplasia related changes (AML-MRC) presented to the outpatient clinic with a positive rapid antigen test for SARS-CoV-2.

The patient had initially received two induction cycles (daunorubicin+ cytarabin[7+3] and idarubicin + cytarabin [within a study]) and had undergone allogenic hematopoietic stem cell transplantation (HSCT). Due to incipient AML-relapse she had undergone one cycle of azacitidine before a second allogenic SCT (complications including: septic shock, reanimation, subarachnoidal hemorrhage). Five months before SARS-CoV-2 infection she had started a maintenance therapy with sorafenib.

She presented to our clinic after a week of fatigue, loss of appetite and nasal congestion. Fever and dyspnea were absent. CT-scan revealed ubiquitous ground glass opacities.

After interdisciplinary case presentation a treatment with remdesivir was started (day 6 after onset of symptoms) and within the German emergency use program the patient received bamlanivimab on day 7 (after onset of symptoms). The therapy was well tolerated and symptoms ceased in the course of the treatment. The patient was released in home quarantine.

On follow up the patient presented with an AML relapse 2 months after the SARS-CoV-2 infection and was started on a palliative regimen with azacitidine and venetoclax.
